# Towards a minimal core dataset for systemic lupus erythematosus studies

**DOI:** 10.1136/lupus-2025-001595

**Published:** 2025-09-22

**Authors:** Stephen McDonald, Jialin Teng, Chengde Yang, Michelle Barraclough, Graciela S Alarcon, Anca D Askanase, Sasha Bernatsky, Ann Elaine Clarke, Nathalie Costedoat-Chalumeau, Qiang Fu, Dafna D Gladman, John G Hanly, Alexandra C Legge, David Isenberg, Kenneth Kalunian, Diane L Kamen, Michelle A Petri, Anisur Rahman, Chuanyin Sun, Ting Li, Murray Urowitz, Alexandre Voskuyl, Daniel J Wallace, Juan Zhang, Ian N Bruce

**Affiliations:** 1 NIHR Manchester Biomedical Research Centre, Manchester Foundation Trust, Manchester Royal Infirmary Kellgren Centre for Rheumatology, Manchester, UK; 2 Rheumatology, HSC Western Health and Social Care Trust, Londonderry, UK; 3 School of Medicine, Department of Rheumatology and Immunology, Shanghai Jiao Tong University, Shanghai, China; 4 Rheumatology and Immunology, Shanghai Jiao Tong University Medical School Affiliated Ruijin Hospital, Shanghai, China; 5 Centre for Musculoskeletal Research, Division of Musculoskeletal and Dermatological Sciences, Faculty of Biology, Medicine and Health, Manchester Academic Health Science Centre, The University of Manchester, Manchester, UK; 6 Marnix E Heersink School of Medicine, The University of Alabama at Birmingham, Birmingham, Alabama, USA; 7 Universidad Peruana Cayetano Heredia, Lima District, Peru; 8 Division of Rheumatology, Columbia University Irving Medical Center, New York City, New York, USA; 9 Division of Rheumatology and Clinical Epidemiology, Research Institute of the McGill University Health Centre, Montreal, Quebec, Canada; 10 Division of Rheumatology, Cumming School of Medicine, University of Calgary, Calgary, Alberta, Canada; 11 Service de Médecine Interne, Centre de référence des maladies auto-immunes et auto-inflammatoires systémiques rares d’Ile-de-France, de l’Est et de l’Ouest, Hopital Cochin, Paris, France; 12 The First Affiliated Hospital of Nanchang Medical College, Jiangxi Provincial People’s Hospital, Nanchang, Jiangxi, China; 13 Department of Medicine, University of Toronto, Toronto, Ontario, Canada; 14 Schroeder Arthritis Institute, Krembil Research Institute, Toronto, Ontario, Canada; 15 Division of Rheumatology, Department of Medicine, Queen Elizabeth II Science Centre & Dalhousie Univeristy, Halifax, Nova Scotia, Canada; 16 Centre for Rheumatology, University College London, London, UK; 17 San Diego School of Medicine, Division of Rheumatology, Autoimmunity and Inflammation, University of California San Diego, La Jolla, California, USA; 18 Medicine, Medical University of South Carolina, Charleston, South Carolina, USA; 19 Division of Rheumatology, Johns Hopkins University School of Medicine, Baltimore, Maryland, USA; 20 Department of Rheumatology, The First Affiliated Hospital of Zhejiang University School of Medicine, Hangzhou, Zhejiang, China; 21 Department of Rheumatology, South Campus, Ren Ji Hospital, School of Medicine, Shanghai Jiaotong University, Shanghai, China; 22 Temerty Faculty of Medicine, University of Toronto, Toronto, Ontario, Canada; 23 Schroeder Arthritis Institute, Toronto Western Hospital, Toronto, Ontario, Canada; 24 Department of Rheumatology and Clinical Immunology, Amsterdam Rheumatology and Immunology Center, Amsterdam, The Netherlands; 25 Rheumatology, Cedars-Sinai Medical Center, West Hollywood, California, USA; 26 Department of Rheumatology, Lanzhou University Second Hospital, Lanzhou, Gansu, China; 27 Faculty of Medicine, Health and Life Sciences, Queen’s University Belfast, Belfast, UK

**Keywords:** Clinical Trial, Lupus Erythematosus, Systemic, Lupus Nephritis

## Abstract

**Objective:**

SLE is a complex, heterogenous autoimmune disease. SLE researchers do not always collect the same data, making comparative studies difficult. We aimed to ascertain what variables SLE clinical researchers commonly collect for SLE research. Our ultimate goal is to generate a minimal core dataset for future SLE studies.

**Methods:**

In 2020, we designed and distributed a questionnaire to members of the Systemic Lupus Erythematosus International Collaborating Clinics (SLICC) as well as additional active research centres in China. Our survey included 26 questions about the types of data that are routinely collected for research. Variables collected by ≥75% of participating respondents were used as a threshold for inclusion.

**Results:**

18 of 36 invited respondents replied (8 from USA/Canada, 5 from China and 5 from Europe). Many key variables in the domains of sociodemographics, SLE specific, comorbidities, baseline haematology/biochemistry/immunology and treatment data were collected by ≥75% respondents including the 1997 American College of Rheumatology (ACR) Classification Criteria (83%), SLE Disease Activity Index-2000 (82%), current treatment (100%), drug name, dose, frequency and start date (75–100%) and complement C3/4 (94%). A range of other items was collected by 50–<75% of respondents including SLICC 2012 Criteria (67%), SLICC/ACR Damage Index (68%) and Short Form Health Survey-36 (53%). Less than 50% of respondents collect certain items including European Alliance of Associations for Rheumatology/ACR 2019 criteria (33%), British Isles Lupus Assessment Group scores (12%) and pneumococcal vaccine status (39%).

**Conclusions:**

The frequency with which an initial set of variables is collected in SLE cohorts globally was identified and can form the basis from which to develop a core minimum dataset for SLE. Further refinement and common definitions will be needed to finalise a minimal core dataset suitable for widespread use.

**WHAT IS ALREADY KNOWN ON THIS TOPIC**

- Despite many SLE cohorts collecting data for studies and registries, there is no consensus on what constitutes a minimum core dataset for SLE cohorts and studies.

**WHAT THIS STUDY ADDS**

- This global survey identified and ranked by frequency of collection, a wide range of variables collected in SLE cohorts and found a relatively small number that are routinely collected by more than 75% of respondents.
- A number of items are also identified that are only collected by less than 50% of respondents globally; some such items will require further consensus to be considered ‘core’ items for use globally.

**HOW THIS STUDY MIGHT AFFECT RESEARCH, PRACTICE OR POLICY**

- These results form the basis from which to develop a core minimum dataset for SLE research.
- Further refinement and common definitions will be needed to enable widespread use.

## Introduction

 SLE is a complex autoimmune disease characterised by variable clinical manifestations and a range of autoantibodies. The prevalence of different disease characteristics, including severity, is influenced by ethnicity and location.^1 2^ Such heterogeneity, combined with different researchers focusing on different aspects of the disease, means that many registries do not collect the same data. This makes comparative studies and multicentre global collaborations difficult. Our aim was to understand what data are routinely collected in different SLE registries/cohorts, with the ultimate goal of generating a core minimal dataset which all centres could collect to facilitate future SLE studies.

## Methods

In 2020, we designed and distributed a questionnaire (online supplemental file S1) to members of the Systemic Lupus Erythematosus International Collaborating Clinics (SLICC) group, as well as similar SLE researchers in China, identified as part of a collaborative network by coauthors (JT, CY). Our survey was comprised of 26 questions about baseline and follow-up data variables that respondents currently collect for their research, including demographics, lifestyle, vaccination, women’s health, SLE diagnosis and disease activity and cumulative organ damage measures, comorbidity, health status questionnaires, blood tests and current/previous treatments. Variables collected were graded by the percentage of respondents collecting each item and grouped in quartiles, that is, ≥75%, 50%–<75%, 25–<50% and <25%. Collection by ≥75% of participating respondents was deemed to be a reasonable threshold for demonstrating a level of natural consensus.^3^ We report percentage responses to the nearest integer and all variables above the 75% threshold as well as notable variables in the lower quartiles below, with the bottom two quartiles combined for ease of reporting.

## Results

We contacted 36 researchers (28 SLICC centres and 8 in China) and 18 (50%) responded to the survey; 8 North American, 5 European and 5 respondents from China.

### Data collected by ≥75% of respondents

This included a range of sociodemographic and lifestyle factors, including racial background, educational attainment, occupation, smoking status and alcohol consumption. Contraception use and pregnancy history (including number of pregnancies, miscarriage, stillbirth and neonatal death) were also collected by 75% or more of respondents. SLE specific data collected by 75% or more of respondents included date and age of diagnosis, disease duration, family history of SLE, American College of Rheumatology (ACR) 1997 classification criteria and SLE Disease Activity Index-2000 (SLEDAI-2K) disease activity measure.^4 5^ Individual comorbidities collected by 75% or more of respondents included diabetes, hypertension, osteoporosis, cerebrovascular disease, ischaemic heart disease, renal disease, infection and malignancy. Obesity and renal-specific data, including renal biopsy results, blood pressure, serum creatinine, urine protein–creatinine ratio and estimated glomerular filtration rate, were also collected by ≥75% of respondents. Complement C3/4, ANAs (by immunofluorescence), extractable nuclear antigens, anticardiolipin antibodies, lupus anticoagulant and anti-beta2 glycoprotein antibodies were measured by ≥75% of respondents. Current therapy was recorded in detail (name, dose, frequency, start date) by 75% or more, whereas previous therapy was commonly restricted to the name of the previous drug only (table 1).

**Table 1:** Data that ≥75% respondents collected

Sociodemographic	Smoking status (94%), Pregnancy history (89%) (number of pregnancies (100%), miscarriage (94%), stillbirth (75%) and neonatal death (75%)), Occupation (88%), Educational attainment (88%), Alcohol consumption (83%), Racial background (78%), Contraception use (75%).
SLE specific	Date at and age of diagnosis (100%), Family history of SLE (94%), ACR 1997 classification criteria (83%), SLEDAI-2000 disease activity measure (82%), Disease duration (78%).
Comorbidity	Diabetes (94%), Hypertension (88%), Bone disease/osteoporosis (88%), Cerebrovascular disease (81%), Obesity (76%), Ischaemic heart disease (75%), Renal disease (75%), Infection (75%), Malignancy (75%).
Renal data	Renal biopsy (100%), Blood pressure (88%), Serum creatinine (88%), Urine protein–creatinine ratio (88%), Estimated glomerular filtration rate (82%).
Baseline bloods	Full blood count (100%), Complement C3/4 (94%), Erythrocyte sedimentation rate (82%), Liver function tests (77%).
Baseline immunology	Anticardiolipin antibody (100%), ANA (indirect Immunofluorescence) (94%), Extractable nuclear antibodies (94%), Lupus anticoagulant (88%), Anti-beta2 glycoprotein antibodies (82%).
Treatment data	Current treatment: Antimalarial (name (88%), dose (94%), frequency (82%), start date (77%)), Intravenous glucocorticoid (name (82%), dose (77%), frequency (82%), start date (77%)), Oral glucocorticoid (name (77%), dose (94%), frequency (82%), start date (77%)), Immunosuppressant (name (94%), dose (94%), frequency (82%), start date (82%)), Biological (name (100%), dose (94%), frequency (82%), start date (82%)), NSAID (name only) (77%). Previous treatment: Antimalarial (name (75%), start date (75%), end date (75%), reason for cessation (81%)), Oral glucocorticoid (name (81%), dose (81%), start date (81%), end date (75%)), Immunosuppressant (name only) (88%), Biological (name only) (88%).

ACR, American College of Rheumatology; NSAID, non-steroidal anti-inflammatory drug; SLEDAI-2000, Systemic Lupus Erythematosus Disease Activity Index-2000.

### Data collected by 50% to <75% of respondents

Sociodemographic factors in this category included ethnicity and income level. SLE specific data collected by 50–<75% of respondents included date of first symptom (67%), Systemic Lupus International Collaborating Clinics (SLICC) 2012 classification criteria (67%),^6^ SLICC/ACR Damage Index (SDI) (68%) and Short Form Health Survey (SF-36) (53%). Other variables collected included influenza vaccination status (54%), menstrual status (69%), hormone replacement therapy use (69%) and history of hysterectomy/sterilisation (69%). Among baseline bloods in this category, serological testing for antibodies to double-stranded-DNA (ds-DNA) by ELISA (71%) and by *crithidia luciliae* test (53%) was included (online supplemental table S2).

### Data collected by <50% of centres

A number of sociodemographic and lifestyle factors were collected by a minority of respondents including physical activity (39%), recreational drug use (33%) and non-binary gender (29%). The European Alliance of Associations for Rheumatology (EULAR)/ACR 2019 classification criteria were collected by a third (33%)^7^ and British Isles Lupus Assessment Group (BILAG)−2004 disease activity measure was collected by 12% of respondents (online supplemental table S3).

## Discussion

In an effort to develop a globally applicable minimal core dataset for SLE, we surveyed SLE researchers to understand what variables they currently collect on patients in their own cohort-based studies. In the absence of any specific published guidance on the key data to collect for SLE research protocols, we used gold standard clinical guidelines such as EULAR 2019 and British Society for Rheumatology 2018 recommendations for the management of SLE to inform the data in the questionnaire.^8 9^ These focus on aspects such as manifestations, medications, cardiovascular disease and damage, all of which we include. We found consensus across researchers on a number of demographic, SLE-specific, therapy-related and comorbidity data that are collected by ≥75% of respondents already. Such items could form the basis of a minimal dataset.

Data on racial background met the required threshold but data on specific ethnic group (56%) did not. The reporting of race/ethnicity across SLE studies is often inconsistent, perhaps reflecting local national policies on race/ethnicity nomenclature, researcher views and/or journal policies.^10^ Since race/ethnicity may influence disease phenotype and SLE outcomes, standardisation of how race/ethnicity is collected could be helpful, particularly for comparisons across countries or other jurisdictions.^11^ The majority of respondents collected data on pregnancy and women’s health data, which is important given the female preponderance of SLE, and the significant pregnancy-related morbidity experienced by women with SLE.

The higher frequency for collection of the ACR 1997 (83%) classification criteria, compared with the SLICC 2012 (67%) and EULAR/ACR 2019 (33%) classification criteria most likely reflects the recent introduction of the latter criteria.^7^ This, however, represents a rapid uptake of the 2019 EULAR/ACR criteria, which is likely to continue over time both in investigator-led studies and commercial trials. Across disease activity measures, SLEDAI-2K (82%) was the most commonly collected measure. In contrast, BILAG-2004 (12%), which is a more comprehensive disease activity measurement, was collected much less frequently. An exercise such as this is not, of course, designed to judge which is the ‘best’ measure for a particular domain. It does, however, demonstrate what the ‘core’ measure should be to support cross-cohort comparisons.

The SDI was collected by 68% of researchers. Given the strong links between damage accrual over time and mortality outcomes,^12^ it is important that damage be considered in any minimal dataset. We did, however, note that many comorbidities that are key components of the SDI, including diabetes, ischaemic heart disease and osteoporosis, were collected by more than 75% of respondents. In doing so, centres are indirectly measuring damage. A formal index such as SDI would be a more valid and reliable approach across centres and so will require wider consultation and a clear statement in spite of these initial findings.

A number of patient-reported outcomes (PROs) have demonstrated content validity.^13^ Our survey noted that the SF-36, although not disease specific, was the most commonly employed PRO measure (53%); other measures (including SLE-specific PROs) were collected by less than half of respondents. This likely reflects that these tools can be time-consuming and are not straightforward to complete even in research clinics. Patients may have different priorities in terms of health outcomes, compared with those of their medical practitioners. There is merit to further assessing the role of PROs in any core dataset.

We found variation in data collection regarding laboratory tests. Complement C3 and C4 levels, as well as the antiphospholipid family of tests, were collected in more than 75% of cohorts. Our questions had separated different measures of anti-dsDNA antibodies, both of which were recorded in <75% of centres; however, either or both were collected by 78% and again illustrate the importance of defining individual domains for assessment.

Regarding therapy, a key observation is that current therapy is collected in detail. In contrast, past therapy is recorded in less detail. This likely reflects the cumbersome nature of historical data collection and incomplete healthcare records, especially in referral centres where past exposures may be less reliably obtained.

Our study has several strengths, including the global spread of centres surveyed which allows us to draw conclusions from centres in very different regions and healthcare systems. In addition, in our study, all respondents had an equal voice in providing item lists. We do acknowledge that the survey is relatively small with only a 50% response rate (18 respondents), which may introduce biases. We further acknowledge that the original data collection was in 2020 and the subsequent delay between the survey being conducted and published; in part due to several personnel changing roles and in part the COVID-19 pandemic. Generally, researchers tend not to change the datasets and case report forms they use in their cohorts and studies frequently so as to maintain consistency in data collection. However, our study shows how a new and important data item, such as the EULAR/ACR 2019 criteria, gets adopted quickly. It also underscores how a core dataset will need to retain a degree of flexibility as new recommendations for indices and outcomes measures emerge. A wider exercise to validate these findings may help refine future consensus building. Opinions from additional research centres outside of Asia, North America and Europe should also be included. We note that several questions may have been ambiguous and will require further clarification (eg, renal function, anti-dsDNA antibodies, gender vs biological sex). This does, however, reinforce the importance of definitions which will also be an important component of the next phase of the project.

## Conclusion

Our survey results have established an initial set of commonly collected variables from a large number of SLE centres across a wide geography. Some researchers may not be collecting certain variables because they were not pertinent to their current research. This does not mean that they could not begin documenting these variables henceforth. These initial results can therefore form the basis of a wider exercise to develop a core minimum dataset for SLE research. Further refinement of core items and common definitions will also be needed to support widespread use.

## Supplementary material

10.1136/lupus-2025-001595 online supplemental file 1

10.1136/lupus-2025-001595 online supplemental file 2

10.1136/lupus-2025-001595 online supplemental file 3

## Data Availability

Data are available upon reasonable request.
