# Supplementary material for: Towards a minimal core dataset for systemic lupus erythematosus studies
Source: Lupus Sci Med. 2025 Sep 22;12(2):e001595. doi: 10.1136/lupus-2025-001595 (PMC12458853; doi:10.1136/lupus-2025-001595)
Supplement: online supplemental file 1 [file lupus-12-2-s001.docx]

**Supplementary Table 1: SLE core minimal dataset survey sent to participants**

**Systemic Lupus Erythematosus core-minimal dataset survey**

Jialin Teng ^1^, Stephen McDonald^2,3^, Ian N Bruce^3,4^

1. Department of Rheumatology, Ruijin Hospital Affiliated to School of Medicine, Shanghai Jiao Tong University.
2. NIHR Manchester BRC, University of Manchester, Manchester, UNITED KINGDOM.
3. The Kellgren Centre, Manchester University Foundation Trust, Manchester Academic Health Sciences Centre, Manchester, UNITED KINGDOM.
4. Arthritis Research UK Centre for Epidemiology, Division of Musculoskeletal and Dermatological Sciences, School of Biological Sciences, Faculty of Biology, Medicine and Health, University of Manchester, UNITED KINGDOM

**Introduction**

Systemic lupus erythematosus (SLE) is an autoimmune disease characterised by a variety of clinical manifestations and a wide profile of autoantibodies. The clinical and serological heterogeneity makes it a great challenge for research. Research questions in the current era require ever larger study populations, which often exceed the number of patients available in individual studies or registries. Hence, combined or pooled analyses are often required.

Through the University of Manchester and SLICC group, we are aiming to determine what data is commonly collected into SLE cohorts. The ultimate goal will be to propose a core-minimal data-set to inform future SLE study. Please answer these questions with reference to your main cohort or key SLE studies.

**Thank you for taking the time to complete the survey.**

**1. Which country do you practice in?**

| Argentina |  |  |
| --- | --- | --- |
| Australia |  |  |
| Canada |  |  |
| Denmark |  |  |
| China |  |  |
| France |  |  |
| Holland |  |  |
| Iceland |  |  |
| Mexico |  |  |
| Singapore |  |  |
| South Korea |  |  |
| Spain |  |  |
| Sweden |  |  |
| Switzerland |  |  |
| Turkey |  |  |
| UK |  |  |
| USA |  |  |
| Other (please specify) |  | |

**Part Ⅰ: Demography**

**2. How do you collect data on race/ethnicity?**

| Ethnic groups |  | Place of birth |  |  |
| --- | --- | --- | --- | --- |
| Racial background |  | Spoken language |  |  |
| Country of origin |  | Other (please specify) |  | |

Please continue over the page….

**3. How do you collect data to classify a patient's socioeconomic status?**

| Educational attainment |  | Social class (high/middle/low) | |  |  |
| --- | --- | --- | --- | --- | --- |
| Current occupation |  | Postcode/Zip code | |  |  |
| Income level |  | Other (please specify) |  | | |

**4. Do you collect any other demographic details?**

|  |
| --- |

**Part Ⅱ: Lifestyle Factors**

**5. What information regarding lifestyle factors do you routinely collect?**

| Smoking status |  | Physical activity | |  |  |
| --- | --- | --- | --- | --- | --- |
| Alcohol consumption |  | Body mass index | |  |  |
| Recreational drug use |  | Other (please specify) |  | | |

**6. Do you collect information regarding vaccinations?**

| Influenza |  | BCG | |  |  |
| --- | --- | --- | --- | --- | --- |
| Haemophilus |  | Varicella status | |  |  |
| Pneumococcal |  | Other (please specify) |  | | |

**7. Do you routinely collect information on woman’s health issues?**

| Menstrual status |  | Hysterectomy/Sterilisation | |  |  |
| --- | --- | --- | --- | --- | --- |
| Contraception use |  |  | |  |  |
| Use of hormone replacement therapy |  | Other (please specify) |  | | |

**8. Do you routinely collect information about pregnancy history?**

Yes

No

Please continue over the page….

**9. If you answered yes to the previous question, what information do you collect pertaining to pregnancy?**

| Number of Pregnancies |  | Neonatal death | |  |  |
| --- | --- | --- | --- | --- | --- |
| Miscarriage |  |  | |  |  |
| Stillbirth |  | Other (please specify) |  | | |

**10. Which family history details do you collect?**

| SLE |  | Cardiovascular risk factors | |  |
| --- | --- | --- | --- | --- |
| Other autoimmune disease |  | Other (please specify) |  | |

**Part Ⅲ: SLE Details**

**11. Regarding SLE, which of the following details do you collect routinely?**

| Date of diagnosis |  | Date of each individual item on SLE  classification criteria met | |  |
| --- | --- | --- | --- | --- |
| Age at diagnosis |  | Date that the SLE classification criteria were met (if different from date of diagnosis) | |  |
| Date of first symptom |  | Disease duration | |  |
| Date of first item on SLE classification criteria met |  | Other (please specify) |  | |

**12. Which of the following SLE classification criteria do you use?**

ACR 1982


ACR 1997

SLICC 2010

EULAR/ACR 2019

Please continue over the page….

**13. Which disease activity measures do you use?**

SLEDAI-2000

ECLAM

SLAM

LAI

BILAG-2004

SELENA-SLEDAI

If you use another assessment, please specify

**14. Do you collect the SLICC/ACR SLE damage index (SDI)?**

Yes


No

**15. Do you specifically collect information on the main organ affected by SLE?**

Yes

No

**Part Ⅳ: Comorbidity**

**16. Separate from the SDI, do you collect additional data on comorbidities?**

Yes


No

Please continue over the page….

**17. If yes, which comorbidities do you focus on?**

| Ischaemic Heart disease |  | Neurological Disease | |  |
| --- | --- | --- | --- | --- |
| Cerebrovascular Disease |  | Psychiatric Disease | |  |
| Hypertension |  | Infection | |  |
| Diabetes Mellitus |  | Bone Disease / Osteoporosis | |  |
| Respiratory Disease |  | Malignancy | |  |
| Renal Disease |  |  |  | |
| Gastrointestinal Disease |  | Other (please specify) |  | |

**Part Ⅴ: Specific detailed organ assessment**

**18. Do you collect information on individual cardiovascular risk factors?**

| Smoking |  | Obesity |  |
| --- | --- | --- | --- |
| Dyslipidemia |  | Diabetes |  |
| Hypertension |  | Chronic anxiety/Depression |  |
| Physical inactivity |  |  |  |

**19. Do you collect specific information on renal disease?**

| Blood pressure |  | Urine albumin creatinine ratio |  |
| --- | --- | --- | --- |
| Serum creatinine |  | Urine protein creatinine ratio |  |
| Renal biopsy |  | Nephrotic syndrome |  |
| Estimated GFR (any formula) |  | 24-hour urinary protein |  |
| Urinary sediment |  | Chronic kidney disease staging |  |
| Urine protein on dipstick |  |  |  |

Please continue over the page….

**Part Ⅵ: Health status**

**20. Which health status questionnaires do you use?**

| SF-36 |  | EuroQoL | |  |
| --- | --- | --- | --- | --- |
| SF-12 |  | Fatigue Severity Scale | |  |
| SLEQoL |  | Lupus Impact Tracker | |  |
| LupusQoL |  | Other (please specify) |  | |

**Part Ⅶ: Blood results**

**21. Do you collect information regarding the following bloods? If yes, please indicate whether** **collected at baseline and follow-up visits?**

| \|  \| Baseline \| Each following visit \| \| --- \| --- \| --- \| \| Full Blood Count \|  \|  \| \| Urea and Electrolytes \|  \|  \| \| Liver Function Tests \|  \|  \| \| Bone Profile \|  \|  \| \| CRP \|  \|  \| \| ESR \|  \|  \| \| Lipids \|  \|  \| \| HbA1C/Glucose \|  \|  \| \| Complement (C3/4) \|  \|  \| \| CH50 \|  \|  \| \| Immunoglobulins \|  \|  \| |  |  |
| --- | --- | --- | --- | --- | --- | --- | --- | --- | --- | --- | --- | --- | --- | --- | --- | --- | --- | --- | --- | --- | --- | --- | --- | --- | --- | --- | --- | --- | --- | --- | --- | --- | --- | --- | --- | --- | --- | --- |

Please continue over the page….

**22. Do you collect information regarding autoantibodies? If yes, please indicate whether collected at baseline and follow-up visits?**

|  | Baseline | Each following visit |
| --- | --- | --- |
| ANA (IIF) |  |  |
| ANA (other) |  |  |
| ENA Profile |  |  |
| ds-DNA (Farr assay) |  |  |
| ds-DNA (ELISA) |  |  |
| ds-DNA (Crithidia) |  |  |
| Lupus anticoagulant |  |  |
| Anticardiolipin antibody |  |  |
| Anti-Beta2 glycoprotein |  |  |

**Part Ⅷ: Treatment**

**23. Do you collect information about current treatment?**

Yes

No

Please continue over the page….

**24. If yes, do you collect any of the following information regarding current treatment?**

|  | Name | | Starting Dose | Current Dose | Frequency | Start Date | |
| --- | --- | --- | --- | --- | --- | --- | --- |
| Oral Glucocorticoids |  | |  |  |  |  | |
| Intra-articular Glucocorticoids |  | |  |  |  |  | |
| Intravenous Glucocorticoids |  | |  |  |  |  | |
| Intramuscular Glucocorticoids |  | |  |  |  |  | |
| Non-Steroidal Anti-Inflammatory Drugs |  | |  |  |  |  | |
| Antimalarials |  | |  |  |  |  | |
| Immunosuppressants |  | |  |  |  |  | |
| Biologics |  | |  |  |  |  | |
| Other (please specify broad categories e.g. antihypertensives) | |  | | | | |  |

**25. Do you collect information about previous treatment?**

Yes

No

Please continue over the page….

|  | Name | | Dose | Frequency | Start Date | End Date | Reason for Cessation | |
| --- | --- | --- | --- | --- | --- | --- | --- | --- |
| Oral Glucocorticoids |  | |  |  |  |  |  | |
| Intra-articular Glucocorticoids |  | |  |  |  |  |  | |
| Intravenous Glucocorticoids |  | |  |  |  |  |  | |
| Intramuscular Glucocorticoids |  | |  |  |  |  |  | |
| Non-Steroidal Anti-Inflammatory Drugs |  | |  |  |  |  |  | |
| Antimalarials |  | |  |  |  |  |  | |
| Immunosupressants |  | |  |  |  |  |  | |
| Biologics |  | |  |  |  |  |  | |
| Other (please specify broad categories e.g. antihypertensives) | |  | | | | | |  |

**26. If yes, do you collect any of the following information regarding previous treatment?**
