# Supplementary material for: Towards a minimal core dataset for systemic lupus erythematosus studies
Source: Lupus Sci Med. 2025 Sep 22;12(2):e001595. doi: 10.1136/lupus-2025-001595 (PMC12458853; doi:10.1136/lupus-2025-001595)
Supplement: online supplemental file 2 [file lupus-12-2-s002.docx]

**Supplemental Table S2: Date collected by 50% to 75% of respondents**

| Sociodemographic | BMI (72.2%),  Menstrual Status (68.8%),  HRT use (68.8%),  Hysterectomy/Sterilisation (68.8%)  Ethnic group (55.6%),  Income level (53%),  Influenza vaccination (53.8%). |
| --- | --- |
| SLE specific | SLICC/ACR SLE damage index (68.4%),  Date of first SLE symptom (66.6%),  Main organ affected by SLE (66.6%),  SLICC 2010 classification criteria (66.6%),  Family History of autoimmune disease (62.5%),  Date of each individual SLE classification item (61.1%),  Date of first SLE classification item (55.6%)  Date full classification met (55.6%),  SF-36 Health questionnaire (53.3%),  SELENA-SLEDAI (52.9%). |
| Comorbidity | Dyslipidaemia (70.6%),  Respiratory disease (56.3%),  Gastrointestinal disease (56.3%),  Neurological disease (56.3%),  Psychiatric disease (50%). |
| Renal Data | CKD stage (64.7%)  Urine Protein on dipstick (58.9%),  Nephrotic Syndrome (58.9%),  24-hour Urinary Protein (58.9%). |
| Baseline bloods | Lipids (70.6%),  Immunoglobulins (70.6%),  CRP (64.7%),  U&E (58.8%),  HbA1C (58.8%). |
| Baseline immunology | Anti-ds-DNA (ELISA) (70.6%),  anti-dsDNA (Crithidia) (52.9%). |
| Treatment data | Current:  NSAID (Dose (70.6%), Frequency (64.7%), Start date (58.8%)),  IM Glucocorticoid (Name (52.9%), Frequency (52.9%)).  Previous:  Immunosuppressant (Dose (62.5%), Frequency (62.5%), Start date (68.8%), End date (68.8%), Reason for cessation (68.8%)),  Biologics (Dose (62.5%), Frequency (62.5%), Start date (62.5%), End date (62.5%), Reason for cessation (68.8%)),  Oral Glucocorticoid (Frequency (62.5%), Reason for cessation (68.8%)),  IV Glucocorticoid (Name (68.8%), Dose (62.5%), Frequency (62.5%), End date (50%)),  Antimalarials (Dose (68.8%), Frequency (68.8%)). |
