# Supplementary material for: Towards a minimal core dataset for systemic lupus erythematosus studies
Source: Lupus Sci Med. 2025 Sep 22;12(2):e001595. doi: 10.1136/lupus-2025-001595 (PMC12458853; doi:10.1136/lupus-2025-001595)
Supplement: online supplemental file 3 [file lupus-12-2-s003.docx]

**Supplemental Table S3: Date collected by < 50% of respondents**

| Sociodemographic | Postcode/ZIP code (41%),  Physical activity (39%),  Pneumococcal vaccination (39%),  Recreational drug use (33%),  Varicella vaccination (31%),  Gender (29%),  Marital Status (29%),  Social class (24%),  BCG vaccination (23%),  Country of origin (23%),  Place of birth (22%),  Medical Insurance (11%),  Year of birth (11%),  Spoken Language (11%),  Menopause age (6%),  Pregnancy status (6%)  Disability (6%),  Race (6%),  Past occupation (6%),  Employment status (6%). |
| --- | --- |
| SLE specific | EULAR/ACR 2019 classification criteria (33%),  Eq5d (13%),  LupusQoL (13%),  LAI (12%),  BILAG-2004 (11%),  SF-12 (7%),  SLEQol (7%),  EuroQol (7%),  Fatigue severity scale (7%),  SLAM (6%),  ACR 1982 classification criteria (6%). |
| Comorbidity | Physical inactivity (29%),  chronic anxiety/depression (18%). |
| Renal data | Urinary sediment (47%),  Urinary albumin-creatinine ratio (12%) |
| Baseline bloods | Bone profile (36%),  CH50 (35%). |
| Baseline immunology | Ds-DNA (Farr assay) (12%) |
| Treatment data | Current:  IM Glucocorticoid (Start date) (41%),  IA Glucocorticoid (Name (47%), Dose (41%), Frequency (47%), Start date (47%)).  Previous:  IV Glucocorticoid (Reason for cessation (38%)),  IM Glucocorticoid (Name (31%), Dose (25%), Frequency (19%), Start date (19%), End Date (19%), Reason for cessation (13%)),  IA Glucocorticoid (Name (31%), Dose (25%), Frequency (19%), Start date (13%), End Date (19%), Reason for cessation (13%)),  NSAID (Name (44%), Dose (38%), Frequency (31%), Start date (25%), End Date (31%), Reason for cessation (25%)). |
